# Prevalence and Associated Factors of the Double Burden of Malnutrition Among Under‐Five Children in Urban Ghana

**DOI:** 10.1002/fsn3.71673

**Published:** 2026-03-22

**Authors:** Deborah Balapou, John Foster Atta‐Doku, Wisdom Kwaku Amuka Achiam, Richard Osei Agjei, Emmanuel Kumah

**Affiliations:** ^1^ Department of Health Administration and Education University of Education, Winneba Winneba Central Region Ghana

**Keywords:** breastfeeding practices, child nutrition, double burden of malnutrition, overnutrition, under‐five children, undernutrition, urban health

## Abstract

The double burden of malnutrition (DBM), reflecting coexisting forms of undernutrition and overnutrition within the same child, is increasingly observed in urbanizing settings. This study assessed the prevalence of child‐level DBM and examined factors associated with DBM among children under five years in urban Ghana. A hospital‐based cross‐sectional study was conducted among 271 mother–child pairs attending a tertiary health facility. Data were collected using a semi‐structured questionnaire and standardized anthropometric measurements, and nutritional status was assessed using the World Health Organization Child Growth Standards. DBM was operationally defined as the coexistence of stunting with either underweight or overweight/obesity within the same child. Multivariable logistic regression was used to examine factors associated with DBM. The prevalence of DBM was 31.37%. Higher odds of DBM were associated with increasing child age (AOR = 1.04; 95% CI, 1.01–1.06), low physical activity (inactive: AOR = 5.73; 95% CI, 1.95–16.86), no history of breastfeeding (AOR = 11.79; 95% CI, 2.68–51.84), and non‐exclusive breastfeeding (AOR = 5.46; 95% CI, 2.13–14.00). Introduction of semi‐solid foods at 6–8 months (AOR = 0.23; 95% CI, 0.10–0.56) and maternal tertiary education (AOR = 0.24; 95% CI, 0.07–0.85) were associated with reduced odds of DBM. DBM is common among under‐five children in urban Ghana and is shaped by early‐life feeding practices, physical activity, and maternal education, underscoring the need for integrated interventions.

## Graphical abstract

Key determinants of the double burden of malnutrition among under‐five children in an urban Ghanaian setting, highlighting risk factors, protective factors, and urban contextual influences.

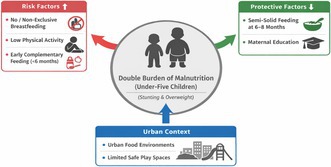

## 1 Introduction

Malnutrition remains a major global public health challenge, encompassing undernutrition, such as stunting, wasting, and micronutrient deficiencies, alongside overnutrition, including overweight and obesity. The coexistence of these contrasting forms within the same population, household, or individual is termed the double burden of malnutrition (DBM) (Shrimpton and Rokx 2012; Tzioumis and Adair 2014). DBM reflects overlapping nutritional challenges increasingly affecting low‐ and middle‐income countries, where rapid social, economic, and dietary transitions drive simultaneous exposure to multiple forms of malnutrition rather than isolated deficits (Downs 2014; Popkin 2015; Prentice 2018).

Globally, the burden of DBM varies by definition and population group. Pooled analyses across 49 low‐ and middle‐income countries estimate a DBM prevalence of approximately 17% when defined as maternal overweight or obesity coexisting with child anemia at the household level (Irache et al. 2022). In sub‐Saharan Africa, stunting remains highly prevalent while childhood overweight is rising, particularly in rapidly urbanizing settings (Onyango et al. 2019; Hawkes et al. 2017). Ghana mirrors this nutrition transition, with persistent child undernutrition alongside increasing exposure to energy‐dense diets and sedentary lifestyles in urban areas. National estimates indicate that concurrent stunting and overweight affect approximately 1.2% of under‐five children (Atsu et al. 2017), while overweight and obesity among women increased by over 130% between 1993 and 2008 (Doku and Neupane 2015). These trends show the growing complexity of malnutrition in urban Ghana.

The consequences of DBM during early childhood are substantial and long‐lasting. Affected children face higher risks of impaired cognitive development, increased susceptibility to infections, poorer educational outcomes, and a greater likelihood of non‐communicable diseases later in life (Prentice 2018; Tzioumis and Adair 2014). At the health‐system level, DBM creates a dual challenge, requiring responses to both undernutrition‐related conditions and obesity‐related chronic diseases, often within resource‐limited environments (Shrimpton and Rokx 2012). Managing these competing demands remains difficult in many LMICs (Hawkes et al. 2017; WHO 2017).

The emergence of DBM results from interacting biological, behavioral, and structural factors. Suboptimal infant and young child feeding practices, including inadequate breastfeeding and inappropriate timing of complementary feeding, remain key drivers of undernutrition (Przyrembel 2012; Martin and Glass 2025). Urbanization has also increased exposure to processed, energy‐dense foods while limiting opportunities for physical activity (Popkin 2015; Romieu et al. 2017). Maternal education, socioeconomic conditions, and access to health information further influence caregiving practices and child nutritional outcomes (Makoka and Masibo 2015; Mbogori et al. 2020). These interacting influences shape early‐life nutritional vulnerability.

Evidence from LMICs highlights early‐life feeding practices, physical activity, and maternal characteristics as factors associated with DBM. Studies from urban African settings document the coexistence of stunting with underweight or overweight among young children, reflecting persistent structural deprivation alongside emerging obesogenic environments (Adeomi et al. 2021; Alaba et al. 2023). Much of the literature, however, emphasizes household‐level DBM, particularly maternal overweight and child undernutrition, rather than child‐level DBM within the same individual (Guevara‐Romero et al. 2022). Systematic reviews similarly report fewer studies on child‐level DBM compared with household or maternal–child dyads, limiting evidence to guide child‐centered interventions in urban contexts (Kosaka and Umezaki 2017).

Globally, nutrition policies increasingly promote “double‐duty actions” that address undernutrition and overnutrition simultaneously, including optimal breastfeeding, improved complementary feeding, and supportive environments for physical activity (WHO 2017; Ruel et al. 2020). Implementation challenges persist in rapidly urbanizing settings, where health systems face limited resources, uneven service coverage, and insufficient context‐specific evidence (Hawkes et al. 2017).

In Ghana, empirical evidence on child‐level DBM in urban settings remains limited. National and subnational surveys describe overall malnutrition trends but provide less insight into the coexistence of multiple forms of malnutrition within individual children and the behavioral and maternal factors shaping risk (Doku and Neupane 2015; Amugsi et al. 2019). The present study addresses this gap by assessing the prevalence of DBM among children under five in an urban Ghanaian setting and examining factors associated with DBM, with emphasis on feeding practices, physical activity, and maternal socio‐demographic characteristics. The findings aim to support integrated, context‐sensitive child nutrition strategies.

## 2 Methods

### 2.1 Study Area and Institution

The study was conducted in Kumasi, the capital of Ghana's Ashanti Region and the country's second‐largest urban center. Located approximately 200 km northwest of Accra, Kumasi covers about 214 km^2^ within the tropical forest belt. According to the 2021 Population and Housing Census, the core city has an estimated population of 443,981, while the Greater Kumasi metropolitan area exceeds 3 million residents (Ghana Statistical Service 2021). Formerly known as the Garden City of West Africa, Kumasi has undergone rapid urbanization, accompanied by infrastructural and spatial planning challenges typical of fast‐growing African cities (Cobbinah et al. 2020).

Komfo Anokye Teaching Hospital (KATH), situated within Kumasi, served as the study site. Established in 1954 and affiliated with the Kwame Nkrumah University of Science and Technology (KNUST), KATH is Ghana's second‐largest tertiary hospital and a major referral center serving the Ashanti, Bono, Ahafo, and northern regions. In addition to serving an urban population, the hospital receives referrals from peri‐urban and rural communities; however, all participants were recruited and received care within an urban tertiary healthcare setting, where service delivery, food environments, and caregiving practices are predominantly urban. The hospital provides specialized maternal and child health services and serves a large and socioeconomically diverse patient population (Komfo Anokye Teaching Hospital 2013, 2025), making it an appropriate setting for investigating child malnutrition in urban Ghana.

### 2.2 Study Design and Sampling Procedure

A hospital‐based cross‐sectional study design was employed using quantitative methods. The study population comprised mother–child pairs attending the outpatient department at KATH. Eligible participants were biological mothers aged 15–49 years attending the outpatient department with their children aged 0–59 months during the study period. Children accompanied by caregivers who were not their biological mothers and mothers who declined to provide informed consent were excluded.

Based on hospital records, an estimated 56 children visited the facility daily. Over the 15‐day data collection period, the study population was projected at 840 (56 × 15) mother–child pairs. The sample size was determined using the Taro Yamane formula: $n=\frac{N}{1+N\left(e^{2}\right).}$

where:

*n* = required sample size.

*N* = population size (840).

e = margin of error (0.05 for 95% confidence level).

$$n=\frac{N}{1+N\left(e^{2}\right)}$$

$$n=\frac{840}{1+840\left(0.05^{2}\right)}$$

*n* = 270.9 ~ 271.

### 2.3 Data Collection Instruments

Data were collected using a semi‐structured questionnaire and standardized anthropometric measurement tools. The selection of independent variables was guided by the United Nations Children's Fund (UNICEF) conceptual framework on the determinants of child malnutrition, which outlines immediate, underlying, and basic causes of malnutrition (Ahmed et al. 2015; Jayatissa 2012; Muhimbula 2024). Variables related to infant and young child feeding practices, physical activity, and maternal socio‐demographic characteristics were further informed by evidence from previous empirical studies conducted in similar settings. The questionnaire was adapted from validated instruments used in earlier studies (Senbanjo et al. 2016; Nti 2008) and modified to reflect the local study context. It included sections on child and maternal socio‐demographic characteristics, obstetric history, environmental factors, infant feeding practices, and dietary behaviors.

Anthropometric data were collected using calibrated equipment in accordance with standard procedures. A digital Seca scale was used to measure the weight of children under two years of age, while a standard adult scale was used for older children and mothers. Length measurements for children under two years were obtained using a recumbent infantometer, and standing height for older children and mothers was measured using a stadiometer or measuring tape. All measurements were recorded to the nearest 0.1 kg or 0.1 cm. Equipment was calibrated daily, and standard zeroing procedures were applied to ensure measurement accuracy and reliability.

### 2.4 Assessment of Nutritional Status

Children's nutritional status was assessed using the 2006 WHO Child Growth Standards. Weight‐for‐age, height‐for‐age, and weight‐for‐height Z–scores were generated using the WHO Anthro package in R. Children with Z–scores below −2 standard deviations from the median were classified as undernourished. Based on WHO classifications, BMI values were categorized into underweight, normal weight, overweight, and obese.

In this study, DBM was defined as the coexistence of stunting (moderate or severe) alongside either overweight/obesity or underweight within the same child. Although conventional definitions of DBM often emphasize the coexistence of stunting and overweight, emerging evidence highlights substantial variability in how DBM is operationalized across studies, particularly in low‐ and middle‐income country contexts. This operational approach aligns with broader conceptual and empirical discussions on DBM definitions, which emphasize the need to capture overlapping forms of malnutrition within individuals rather than relying on a single standardized classification (Davis et al. 2020). This broader operationalization reflects emerging evidence on compounded malnutrition forms that impair child development in resource‐limited settings (Dembélé et al. 2018; Adeomi et al. 2021).

### 2.5 Validity of Data Collection Instruments

Content validity was addressed through a review of the questionnaire and research protocol by a public health expert and the Research Department at KATH. Revisions were made based on expert feedback to enhance contextual appropriateness and clarity. A pilot test was conducted among a small sample of mother–child pairs to assess question sequence, comprehension, and flow.

### 2.6 Data Collection

Data collection was conducted over 15 consecutive working days from 19th June to 7th July 2023 at the hospital's outpatient department. Trained research assistants administered the questionnaires and performed anthropometric measurements under daily supervision to ensure adherence to study protocols. Completed questionnaires were reviewed each day for accuracy and completeness before data entry and analysis.

### 2.7 Data Analysis

Data analysis was conducted using R version 4.5.0 and STATA version 18. Descriptive statistics were used to summarize socio‐demographic and nutritional characteristics. The prevalence of the double burden of malnutrition (DBM) was calculated as the proportion of children exhibiting coexisting indicators of undernutrition (e.g., stunting or underweight) and overnutrition (e.g., overweight or obesity). Chi‐square tests were used to examine bivariate associations between DBM and child and maternal characteristics. Variables with a *p*‐value < 0.25 in the bivariate analysis were entered into a binary logistic regression model to identify factors independently associated with DBM. Multicollinearity was assessed prior to model estimation using Variance Inflation Factors (VIF). All variables had VIF values below the commonly accepted threshold of 10, with a mean VIF of 2.54, indicating no evidence of multicollinearity (see Table S1). Adjusted odds ratios (AORs) and 95% confidence intervals (CIs) were computed and presented to support interpretation.

## 3 Results

### 3.1 Children's Sociodemographic Characteristics

A total of 271 under‐five children were recruited at Komfo Anokye Teaching Hospital. The mean age was 20.63 months (SD ±1.08), with the largest group being those under six months (26.94%). Males comprised 54.98% of the sample. Most children were from Christian households (84.87%), and over half (56.09%) were reported as very active. Regarding fathers' education, the majority had either secondary (36.53%) or tertiary education (35.79%). Please refer to Table 1 for more details.

**TABLE 1:** Children's socio‐demographic characteristics (*n* = 271).

*Child Socio‐demographic Characteristics*	*Frequency (n)*	*Percentage (%)*
***Child's Age Category***	Mean age in Months: 20.63 ± 1.08	
< 6 Months	73	26.94
6–12 Months	55	20.30
13–24 Months	61	22.51
25–36 Months	36	13.28
37–59 Months	46	16.97
***Child's Sex***		
Male	149	54.98
Female	122	45.02
***Child's Religion***		
Christian	230	84.87
Islam	40	14.76
Traditional	1	0.37
***Child's Birth Order***		
1st	75	27.68
2nd–4th	166	61.25
5th–7th	30	11.07
***Child's Daily Activities***		
Very Active	152	56.09
Active some of the time	88	32.47
Not Active	31	11.44
***Father's Educational Level***		
No Formal Education	12	4.43
Basic	56	20.66
Secondary	99	36.53
Postsecondary	7	2.58
Tertiary	97	35.79

### 3.2 Children's Dietary Practices

Table 2 presents the feeding practices of the children. The vast majority (96.31%) had ever been breastfed. Breastfeeding was initiated within six hours of birth in 55.72% of cases, with 39.48% initiating within the first hour and 4.80% after six hours. Only 40.59% of the children were exclusively breastfed for six months. Formula feeding was common; 70.11% received formula, with 49.47% of those introduced before six months. Regarding semi‐solid food introduction, 53.14% began between six and eight months, 41.33% before six months, and 5.54% after eight months.

**TABLE 2:** Children's dietary practices (*n* = 271).

Variable	Frequency (*n*)	Percentage (%)
***Breastfeeding (Ever)***		
Yes	261	96.31
No	10	3.69
***Initiation of Breastfeeding***		
< 1 Hour	107	39.48
> 6 Hours	13	4.80
< 6 Hours	151	55.72
***Exclusive Breastfeeding (6 Months)***		
Yes	110	40.59
No	161	59.41
***Using Formula Feed***		
Yes	190	70.11
No	81	29.89
***Introduced formula feed before six months (n = 190)***		
Yes	94	49.47
No	96	50.53
***Age of Semi‐solid Food Introduction***		
< 6 Months	112	41.33
6–8 Months	144	53.14
> 8 Months	15	5.54

### 3.3 Mothers' Sociodemographic Characteristics

The sociodemographic profile of the mothers is presented in Table 3. The majority were aged 31–39 years (53.14%), followed by 20–30 years (30.63%), 40 years and above (14.39%), and below 20 years (1.85%). Most mothers were married (84.13%) and resided in urban areas (89.30%), while a small proportion (10.70%) resided outside the urban core due to the hospital's referral catchment. Regarding education, 35.79% had a tertiary education, 32.84% had basic education, 25.09% had secondary education, and a smaller proportion had postsecondary (1.11%) or no formal education (5.17%). In terms of employment, 80.07% were employed. Physical activity levels were low, with 84.50% reporting no weekly exercise, 14.39% exercising one to three days per week, and only 1.11% exercising more than four days weekly.

**TABLE 3:** Mothers' socio‐demographic characteristics (*n* = 271).

	*Frequency (n)*	*Percentage (%)*
***Mother's Age***		
Below 20 years	5	1.85
20–30 years	83	30.63
31–39 years	144	53.14
40 and above	39	14.39
***Marital Status***		
Married	228	84.13
Single	43	15.87
***Mother's Educational Level***		
No Formal Education	14	5.17
Basic	89	32.84
Secondary	68	25.09
Postsecondary	3	1.11
Tertiary	97	35.79
***Residence***		
Outside Urban Core	29	10.70
Urban	242	89.30
***Employment Status***		
Employed	217	80.07
Not Employed	54	19.93
***Exercise Frequency (Weekly)***		
Never	229	84.50
1–3 Days	39	14.39
More than 4 Days	3	1.11

### 3.4 Nutritional Status of Children

Table 4 presents the nutritional classifications. The majority of children (82.29%) were underweight, 13.28% had normal BMI, and 4.43% were overweight or obese. For height‐for‐age, 63.10% were classified as normal, 19.93% as stunted, and 16.97% as severely stunted. Based on the study's definition, the double burden of malnutrition (underweight–stunting or overweight–stunting) was identified in 31.37% of the children. Given the relatively low proportion of overweight children, it is likely that a significant portion of these cases involved underweight coexisting with stunting.

**TABLE 4:** Distribution of children by BMI, stunting, and double burden of malnutrition (*n* = 271).

	Frequency (*n*)	Percentage (%)
***BMI Category***		
Normal	36	13.28
Underweight	223	82.29
Overweight/Obese	12	4.43
***Stunting Category***		
Normal	171	63.10
Stunted	54	19.93
Severely Stunted	46	16.97
***Double Burden of Malnutrition***		
Yes	85	31.37
No	186	68.63

### 3.5 Bivariate Associations With the Double Burden of Malnutrition

Chi‐square tests were conducted to examine associations between the double burden of malnutrition (DBM) and various child and maternal characteristics (Table 5). A significant association was found between DBM and the child's activity level (χ^2^ = 9.374, *p* = 0.009), breastfeeding history (*χ* ^2^ = 3.955, *p* = 0.047), and use of formula feed (*χ* ^2^ = 7.529, *p* = 0.006). Mother's education was also significantly associated with DBM (*χ* ^2^ = 11.846, *p* = 0.019). Other factors, including the child's sex, birth order, maternal age, marital status, residence, employment, and physical activity, were not significantly associated.

**TABLE 5:** Association between DBM and child/maternal characteristics (*n* = 271).

	*Double Burden malnutrition*			*χ* ^*2*^	*p*
	*Yes*	*No*	*Total*		
***Child's Sex***					
Male	101	48	149	0.111	0.739
Female	85	37	122		
***Birth Order***					
1	49	26	75	0.719	0.698
2–4	115	51	166		
5–7	22	8	30		
***Child's Daily Activities***					
Very Active	114	38	152	9.374	0.009***
Active sometimes	57	31	88		
Not Active	15	16	31		
***Father's Education***					
No formal	8	4	12	4.986	0.289
Basic	34	22	56		
Secondary	65	34	99		
Postsecondary	6	1	7		
Tertiary	73	24	97		
***Breastfed Ever***					
Yes	182	79	261	3.955	0.047**
No	4	6	10		
***Initiation of Breastfeeding***					
< 1 h	71	36	107	2.164	0.339
< 6 h	108	43	151		
> 6 h	7	6	13		
***Exclusive Breastfeeding (6 Months)***					
Yes	82	28	110	3.005	0.083*
No	104	57	161		
***Have you/will you introduce formula feed?***					
Yes	140	50	190	7.529	0.006***
No	46	35	81		
***Introduced formula feed before 6 months (n = 190)***					
Yes	66	28	94	1.156	0.282
No	74	22	96		
***Age of Semi‐solid Food Introduction***					
< 6 months	74	38	112	2.834	0.242
6–8 months	104	40	144		
> 8 months	8	7	15		
***Mother's Age***					
< 20 years	2	3	5	2.126	0.547
20–30 years	57	26	83		
31–39 years	101	43	144		
40+ years	26	13	39		
***Marital Status***					
Married	161	67	228	2.615	0.106
Single	25	18	43		
***Mother's Education***					
No formal	6	8	14	11.846	0.019***
Basic	63	26	89		
Secondary	41	27	68		
Postsecondary	1	2	3		
Tertiary	75	22	97		
***Residence***					
Outside Urban Core	19	10	29	0.147	0.702
Urban	167	75	242		
***Employment***					
Yes	153	64	217	1.773	0.183
No	33	21	54		
***Exercise (Weekly)***					
Never	158	71	229	1.757	0.415
1–3 days	27	12	39		
> 4 days	1	2	3		

*** *p* < 0.01.

** *p* < 0.05**.

* *p* < 0.1.

### 3.6 Factors Associated With the Double Burden of Malnutrition

Table 6 presents the bivariable and multivariable logistic regression results for factors associated with the double burden of malnutrition (DBM). In the adjusted analysis, increasing child age was associated with higher odds of DBM (AOR = 1.04; 95% CI, 1.01–1.06). Compared with very active children, those who were active some of the time (AOR = 2.18; 95% CI, 1.01–4.68) and inactive children (AOR = 5.73; 95% CI, 1.95–16.86) had higher odds of DBM. Feeding practices were significantly associated with DBM. Children who had never been breastfed (AOR = 11.79; 95% CI, 2.68–51.84) or were not exclusively breastfed for six months (AOR = 5.46; 95% CI, 2.13–14.00) had increased odds of DBM. Children who did not receive formula feeding showed borderline evidence of higher odds of DBM after adjustment (AOR = 2.04; 95% CI, 0.99–4.18). Introduction of semi‐solid foods at 6–8 months was associated with reduced odds of DBM (AOR = 0.23; 95% CI, 0.10–0.56), while introduction after eight months was not significant. Maternal tertiary education was protective (AOR = 0.24; 95% CI, 0.07–0.85), whereas marital status and maternal employment showed no independent associations.

**TABLE 6:** Bivariable and multivariable logistic regression analysis of factors associated with the double burden of malnutrition among child/maternal characteristics (*n* = 271).

Variable	Crude OR (95% CI)	*p*	AOR (95% CI)	*p*
**Age (months)**	1.00 (0.99–1.01)	0.943	1.04 (1.01–1.06)	0.001
**Child's daily activities**				
Very active^**b**^				
Active some of the time	1.63 (0.92–2.89)	0.093	2.18 (1.01–4.68)	0.046
Not active	3.20 (1.45–7.08)	0.004	5.73 (1.95–16.86)	0.002
***Have/are you breastfeeding***				
Yes^**b**^				
No	3.46 (0.95–12.58)	0.060	11.79 (2.68–51.84)	0.001
***Exclusive breastfeeding for six months***				
Yes^**b**^				
No	1.61 (0.94–2.75)	0.084	5.46 (2.13–14.00)	< 0.001
**Using formula feed**				
Yes^*b*^				
No	2.13 (1.23–3.68)	0.007	2.04 (0.99–4.18)	0.052
***Age of introducing semi‐solid feed***				
< 6 months^**b**^				
6–8 months	0.75 (0.44–1.28)	0.289	0.23 (0.10–0.56)	0.001
> 8 months	1.70 (0.57–5.05)	0.337	0.51 (0.13–2.07)	0.348
**Marital status**				
Married^**b**^				
Single	1.73 (0.89–3.38)	0.109	1.47 (0.67–3.24)	0.341
**Educational level**				
No formal education^**b**^				
Basic	0.31 (0.10–0.98)	0.046	0.29 (0.08–1.05)	0.059
Secondary	0.49 (0.15–1.58)	0.235	0.44 (0.12–1.57)	0.203
Postsecondary	1.50 (0.11–20.68)	0.762	0.98 (0.05–17.82)	0.990
Tertiary	0.22 (0.07–0.70)	0.011	0.24 (0.07–0.85)	0.027
**Employment status**				
Yes^**b**^				
Not employed	1.52 (0.82–2.83)	0.185	1.59 (0.78–3.26)	0.206

Abbreviations: AOR, adjusted odds ratio; CI confidence interval; Crude OR, unadjusted odds ratio.

^b^ Reference category.

## 4 Discussion

The coexistence of undernutrition and overnutrition observed in this study reflects broader nutritional transitions seen in urbanizing low‐ and middle‐income countries, where rising overweight trends increasingly accompany persistent stunting and wasting (Tzioumis and Adair 2014; Mahmudiono et al. 2019). Despite a relatively low proportion of overweight or obese children, the prevalence of DBM was considerably higher, suggesting that many cases involved underweight–stunting combinations. This pattern suggests a context‐specific form of nutritional duality, shaped more by chronic deprivation than dietary excess. Clarifying these underlying patterns through disaggregated DBM classifications is essential for identifying context‐specific vulnerabilities and developing more precise, responsive interventions.

In comparison with recent studies from urban African settings, the prevalence of child‐level DBM observed in this study aligns with evidence from contexts undergoing rapid nutrition transitions. Studies from urban Nigeria, Kenya, and South Africa have similarly reported substantial coexistence of stunting with either underweight or overweight among young children, reflecting persistent structural deprivation alongside emerging obesogenic exposures (Adeomi et al. 2021; Alaba et al. 2023; Mbogori et al. 2020). However, the DBM prevalence observed in Kumasi is higher than estimates reported in some East African urban contexts, where overweight–stunting combinations dominate more clearly, suggesting that chronic undernutrition remains a stronger driver of nutritional duality in urban Ghana. This divergence highlights the importance of contextualizing DBM within local food systems, caregiving norms, and socioeconomic gradients rather than assuming uniform transition trajectories across urban Africa.

Physical activity emerged as a critical behavioral factor associated with the double burden of malnutrition. Children who were reported to be inactive or only intermittently active were significantly more likely to exhibit coexisting indicators of under‐ and overnutrition. The pattern reflects broader urban realities in Ghana and other sub‐Saharan African settings, where safe, accessible play spaces are limited, and children's routines are increasingly shaped by sedentary behaviors such as screen time. In environments characterized by spatial constraints and safety concerns, opportunities for regular movement and physical engagement may be compromised, contributing to poor nutritional outcomes (Aryeetey et al. 2017; Santos et al. 2023). These findings highlight the importance of child‐centered urban planning and interventions that encourage daily physical activity.

Feeding practices were strongly associated with DBM. Children who had never been breastfed or were not exclusively breastfed for the first six months showed higher odds of DBM. Exclusive breastfeeding supports healthy metabolic development, enhances immune function, and is protective against both stunting and excess weight gain (Azad et al. 2018; Shipp et al. 2024). Disruptions in breastfeeding, whether due to limited maternal knowledge, work constraints, or sociocultural practices, may therefore increase vulnerability to malnutrition in both forms. Importantly, this study provides context‐specific evidence that the timing of complementary feeding also plays a critical role in shaping DBM risk. The introduction of semi‐solid foods at 6–8 months, consistent with WHO complementary feeding guidance (Przyrembel 2012; Martin and Glass 2025), was associated with significantly reduced odds of DBM, while both early introduction (< 1.08 6 months) and delayed transition (> 8 months) appear to reflect broader caregiving and structural constraints that heighten nutritional vulnerability in urban Ghanaian settings.

Related to these feeding dynamics, not receiving formula feeding showed borderline evidence of higher odds of DBM after adjustment. This pattern should not be interpreted as promoting formula feeding; instead, it may reflect feeding transitions in which breastfeeding is insufficient or interrupted, and children are introduced early to nutrient‐poor complementary foods. Similar findings in low‐resource settings suggest that compounded nutritional risk is often driven by suboptimal transition feeding practices rather than formula use per se (Silva‐Neto et al. 2024; Mahmudiono et al. 2019). These results reinforce the importance of supporting adequate and timely infant feeding transitions in line with double‐duty actions for nutrition (WHO 2017).

Maternal education significantly influenced the likelihood of children experiencing the double burden of malnutrition. Those whose mothers had a tertiary education were substantially less affected, suggesting that education equips women with the skills to make informed health and nutrition decisions, navigate healthcare systems, and critically assess child feeding recommendations. This supports prior evidence that maternal education enhances not only health literacy but also economic independence and responsiveness to early signs of nutritional distress (Makoka and Masibo 2015). Even mothers with only basic education showed marginally reduced odds of having children with DBM, indicating that any formal education can confer benefits. These findings highlight the need to invest in female education as a long‐term strategy for improving child nutrition and addressing structural drivers of malnutrition in urban Ghana.

The patterns observed in this study point to deeper systemic and structural imbalances shaping child nutrition in urban Ghana. The double burden of malnutrition does not arise solely from individual choices but is embedded in broader inequalities involving access to nutritious foods, reliable health information, and quality healthcare services. Similar to trends observed across SSA, the coexistence of undernutrition and overweight often affects urban poor populations who face intersecting challenges such as food insecurity, limited maternal education, and constrained living environments (Oladeji et al. 2025; Nyanhanda et al. 2023; Seifu et al. 2024). Interventions must therefore be multifaceted, promoting appropriate feeding, encouraging physical activity, and addressing broader social and economic barriers.

This study advances the evidence base by focusing on child‐level DBM, a relatively underexplored dimension of malnutrition in Ghana. The integration of behavioral, maternal, and demographic data offers a holistic view of DBM and supports the need for double‐duty actions that address under‐ and overnutrition simultaneously. Public health strategies should prioritize early‐life nutrition, enhance maternal support systems, and strengthen local delivery of targeted nutrition and physical activity programs, especially in rapidly urbanizing settings.

## 5 Conclusion

This study found a high burden of DBM among children under five in an urban Ghanaian setting. DBM was significantly associated with increasing child age, low levels of physical activity, absence of breastfeeding, and non‐exclusive breastfeeding. In contrast, the timely introduction of semi‐solid foods and higher maternal education were associated with lower odds of DBM. These findings highlight the importance of strengthening breastfeeding promotion, supporting appropriate complementary feeding practices, encouraging regular physical activity among young children, and improving maternal education as key strategies for reducing DBM among children under five in urban Ghana.

## 6 Limitations

The cross‐sectional design limits causal interpretation of associations with DBM. The reliance on convenience sampling at a single tertiary facility may introduce selection bias, as the study population may not be representative of the broader urban under‐five population in Ghana. Maternal self‐reports of feeding practices and physical activity may also be subject to recall and social desirability bias. Another limitation is the narrow focus on anthropometric indicators; micronutrient deficiencies, food insecurity, and household socioeconomic status were not assessed. These factors could significantly influence nutritional outcomes and should be explored in future research.

## Author Contributions

**Deborah Balapou:** conceptualization, methodology, data curation, investigation, writing – original draft, project administration, resources. **Emmanuel Kumah:** conceptualization, methodology, validation, writing – review and editing, supervision. **Wisdom Kwaku Amuka Achiam:** conceptualization, methodology, software, data curation, formal analysis, project administration, investigation, writing – original draft, writing – review and editing, resources. **Richard Osei Agjei:** conceptualization, writing – review and editing, supervision, validation, methodology. **John Foster Atta‐Doku:** conceptualization, methodology, software, investigation, formal analysis, writing – original draft, writing – review and editing, project administration, data curation, resources.

## Funding

The authors have nothing to report.

## Disclosure

No generative artificial intelligence tools were used to generate, analyze, or interpret study data or to draft the scientific content of this manuscript. Language assistance tools were limited to grammar and reference formatting checks and did not alter the authors' conclusions.

## Ethics Statement

Ethical clearance for this study was obtained from the Komfo Anokye Teaching Hospital Institutional Review Board (KATH/IRB/2023). Written informed consent was obtained from all participating mothers after the purpose and procedures of the study were explained. Participation was voluntary, and confidentiality was ensured through anonymized data collection and secure data storage.

## Consent

The authors have nothing to report.

## Conflicts of Interest

The authors declare no conflicts of interest.

## Supporting information


**Table S1:** VIF Values for Predictors in the Logistic Regression Model (*n* = 271).

## Data Availability

The data that support the findings of this study are available from the corresponding author upon reasonable request.
