# Supplementary material for: Prevalence and Associated Factors of the Double Burden of Malnutrition Among Under‐Five Children in Urban Ghana
Source: Food Sci Nutr. 2026 Mar 22;14(3):e71673. doi: 10.1002/fsn3.71673 (PMC13093786; doi:10.1002/fsn3.71673)
Supplement: Supplementary file 1 — Table S1: VIF Values for Predictors in the Logistic Regression Model (n = 271). [file FSN3-14-e71673-s001.docx]

**Table S1: VIF Values for Predictors in the Logistic Regression Model (n=271)**

| **Variable** | **VIF** | **1/VIF** |
| --- | --- | --- |
| ***Age of child (in months)*** | 1.61 | 0.621 |
| ***Child’s Daily Activities*** |  |  |
| Active some of the time | 1.55 | 0.644 |
| Not active | 1.64 | 0.608 |
| ***Currently Breastfeeding (No)*** | 1.10 | 0.910 |
| ***Exclusive Breastfeeding (No)*** | 2.78 | 0.360 |
| ***Using Formula Feed (No)*** | 1.54 | 0.649 |
| ***Age of Semi-Solid Food Introduction*** |  |  |
| 6–8 months | 3.41 | 0.293 |
| > 8 months | 1.54 | 0.649 |
| ***Marital Status (Single)*** | 1.13 | 0.882 |
| ***Educational Level*** |  |  |
| Basic | 5.22 | 0.192 |
| Secondary | 4.61 | 0.217 |
| Postsecondary | 1.26 | 0.796 |
| Tertiary | 5.45 | 0.183 |
| ***Employment Status (No)*** | 1.09 | 0.914 |
| Mean VIF | 2.54 | |
